# The effect of ‘Out of hours surgery Service’ in Israel on hip fracture fixation outcomes: a retrospective analysis

**DOI:** 10.1186/s13584-017-0150-7

**Published:** 2017-07-15

**Authors:** Yaniv Keren, Sybil Sailofsky, Doron Keshet, Michal Barak

**Affiliations:** 1 The Department of Orthopedic Surgery, Rambam Health Care Campus and the Bruce Rappaport Faculty of Medicine, Technion-Israel Institute of Technology, Haifa, Israel; 2 The Bruce Rappaport Faculty of Medicine, Technion-Israel Institute of Technology, Haifa, Israel; 3 The Department of Orthopedic Surgery, Rambam Health Care Campus, Haifa, Israel; 4 The Department of Anesthesiology, Rambam Health Care Campus and the Bruce Rappaport Faculty of Medicine, Technion-Israel Institute of Technology, POB 9602, Haifa, 31096 Israel

**Keywords:** Hip fracture, Surgery, Post-operative mortality, Osteoporosis

## Abstract

**Background:**

‘Out of Hours Surgery Service’ (OHSS) was implemented in Israel, amongst other reasons, in order to reduce the time interval between hospital admission and surgery and consequently improve outcomes. The OHSS is currently operated in the public hospitals in Israel. In this study we compared the data of patients before and after OHSS implementation to determine its efficacy in improving patient care.

**Methods:**

This is a retrospective observational study of 792 adult patients who underwent hip fracture surgery between 2002 and 2007 in a single hospital. The study population included two groups: patients that were operated before the implementation of the OHSS (2002–2004) and after the implementation of the OHSS (2005–2007). Data regarding all patients was collected using the institution’s computer program. The following variables were analyzed: patients’ demographics, time interval from hospitalization to surgery, causes for delaying surgery, post-operative length of hospitalization and mortality.

**Results:**

Patients in the post-OHSS group had more illnesses and higher ASA classification than those in the pre-OHSS group. The post-OHSS group had a significantly decreased length of stay in the hospital before and after the surgery. After adjusting for ASA score and age, the post-OHSS group was found to have decreased post-operative hospitalization and lower post-operative mortality. Surgery was delayed in pre-OHSS period mainly due to operating rooms unavailability.

**Conclusion:**

Implementation of OHSS facilitated operating room availability, thus early operation and reduced post-operative mortality. In accordance with other studies, patient’s outcome is greatly influenced by the time from admission to hip fracture surgery.

## Background

Osteoporotic hip fracture is the most common orthopedic injury in the elderly population and a major health problem worldwide [1, 2]. Its incidence has been rising steadily, mainly due to increased life expectancy [2, 3], and this trend is expected to continue in the future. There were 1.6 million osteoporotic hip fractures throughout the world in 2000 [2] and it has been estimated that the number of hip fractures in 2025 will be 2.6 million and in 2050 will be 4.5 million [4]. In Israel, the incidence of hip fractures more than doubled in 20 years, especially in the over-75 year old age group [5].

The incidence of hip fracture begins to rise at age 50 and peaks in the eighth decade of life, mainly due to osteoporosis at that age [2, 3]. Following fracture, mortality ranges from 4% at 1 month to 33% at 1 year, with approximately 70% of deaths at 1 year attributed to the hip fracture [6, 7]. Surgery is the selected method used to treat hip fractures. Patients who are not operated have a much higher mortality rate [8, 9]. Efforts to reduce the morbidity and mortality associated with hip fracture have focused on 2 main areas: fracture prevention through falls reduction and osteoporosis treatment [10, 11] and improved timeliness of surgery.

Previous studies have debated the most beneficial time period in which hip surgery should be performed to further reduce mortality, with a suggestion that operating the patient within 48 h of injury may reduce the mortality, as well as the complications and hospital stay of the patient [12–19]. Early surgery minimizes the length of time a patient is confined to bed rest, thereby reducing the risk of associated complications, such as pressure sores, deep vein thrombosis and urinary tract infections. On the other hand, delay before the surgery provides the opportunity to optimize patients’ medical status, thereby decreasing the risk of perioperative complications. The effect of early surgery on hip fracture outcomes has received considerable study, and although research suggests that early surgical treatment of these fractures leads to better patient outcomes, studies to date are inconclusive [12–15, 20–22]. Time-to-hip-fracture-surgery standards remain a subject of much debate because supporting evidence is limited by methodology and selection bias, as well as geographic and health-care-systems differences between studies [14, 15]. Thus it may be erroneous to conclude about health care management based on studies that were conducted in other countries.

In order to reduce time to surgery, an ‘Out of Hours Surgery Service’ (OHSS) was implemented by the Ministry of Health in Israel. This service uses hospital facilities and medical professionals after hours, with an additional payment per patient to all caretakers (surgeons, anesthesiologists, and nurses) and to the hospital [23]. Payment was made only if the patient was operated within 48 h from admission, hence the incentive for early treatment by all involved. The OHSS system is currently operated in the public health system in Israel.

The aim of this study was to determine whether the implementation of the OHSS in Israel resulted in reduced waiting time before surgery, and investigate the causes for delaying surgery in cases that were postponed. In addition we tested the theory that patients in the post-OHSS period benefit in terms of post-operative outcomes, such as length of stay after surgery and mortality.

## Methods

This retrospective observational study was approved by the Ethic Committee of Rambam Health Care Campus (approval number: 0287-15-RMB). The study included data from all adult patients who underwent emergent hip fracture surgery between 01/01/2002 to the end of 2007. Patients that were included had hip surgery as their only surgical treatment at that hospitalization. The study population was divided into two groups: patients who were operated before the implementation of the OHSS (2002–2004) and patients who were operated after the implementation of the OHSS (2005–2007). All the patients, pre- and post-OHSS, were operated between 3:00 pm and 8:00 am of the next day. The surgical, anesthetic and post-operative care were the standard care at that time.

Data regarding all patients was collected from the computer "Prometheus" program used at the Rambam Health Care Campus, which records all patient information. Many variables were collected and analyzed. These include:

- Demographic data: patient's age, gender
- Data regarding the patient’s medical status: number of medications, number of illnesses, American Society of Anaesthesiologists (ASA) physical status classification (from 1- the better status, to 5- the worse) [24]
- Data regarding the operation: total time of operation from admission to operating room to patient’s transfer to recovery room, and actual operation time
- Outcomes: duration of hospitalization, length of stay before surgery and length of stay after surgery, and post-operative mortality
- In patients that were operated more than 48 h after admission, what was the cause for the delaying surgery

### Statistical analysis

The baseline characteristics of the pre-OHSS and post-OHSS groups were compared using a chi square test. Binary logistic regression was used for the calculation of the odds ratios (OR) with 95% confidence intervals (CI) and p values in bivariate analysis of factors for time interval from hospitalization to surgery ≤ 48 h, length of hospitalization after surgery > 7 days, and post-operative mortality (after 30 days and 1 year).

Candidates for multivariate analysis were chosen according to *p* value < 0.1.

Multinomial logistic regression analysis was performed to assess the relation of the OHSS and all other relevant variables with the outcomes stated above.

The area under the receiver operating characteristic (ROC) curve was used as a measure of models discrimination. The Hosmer-Lemeshow goodness-of-fit statistic was calculated. Two-tailed *p* values of 0.05 or less were considered as statistically significant. Statistical analyses were performed using SPSS (Statistics Products Solutions Services) 21.0 software for Windows.

## Results

Data from 792 patients were included in this study, 335 from the pre-OHSS period and 457 from the post-OHSS period. Chi square analysis of demographic and clinical characteristics of patients reveals several differences between pre-OHSS and post-OHSS groups (Table 1). While age and gender were similar in both groups, the post-OHSS had significantly more illnesses, were using more medications, and more patients were with high ASA classifications.

**Table 1:** Data regarding the demographic characteristics of the patients and the operation

Characteristic	TOTAL	Pre-OHSS	Post-OHSS	*P* value
Age				
< 70	170 (21%)	71 (21%)	99 (22%)	0.771
70–79	195 (25%)	80 (24%)	115 (25%)	
80–89	325 (41%)	136 (41%)	189 (41%)	
90+	102 (13%)	48 (14%)	54 (12%)	
Gender				
Female	564 (71%)	238 (71%)	326 (71%)	0.937
Male	228 (29%)	97 (29%)	131 (29%)	
Medications				
0	169 (21%)	86 (26%)	83 (18%)	<0.0001
1	73 (9%)	46 (14%)	27 (6%)	
2	98 (12%)	42 (13%)	56 (12%)	
3	452 (57%)	161 (48%)	291 (64%)	
Illnesses				
0	115 (15%)	54 (16%)	61 (13%)	0.019
1	137 (17%)	66 (20%)	71 (16%)	
2	162 (20%)	77 (23%)	85 (19%)	
3+	378 (48%)	138 (41%)	240 (53%)	
ASA^a^				
1	56 (7%)	24 (7%)	32 (7%)	<0.0001
2	244 (31%)	135 (40%)	109 (24%)	
3	385 (49%)	141 (42%)	244 (53%)	
4	107 (14%)	35 (10%)	72 (16%)	
LOS Before^b^				
< 24 h	306 (39%)	112 (33%)	194 (42%)	<0.0001
24–48 h	242 (31%)	68 (20%)	174 (38%)	
48+ hours	244 (31%)	155 (46%)	89 (19%)	
Total duration^c^				
< 51 min	475 (60%)	172 (51%)	303 (66%)	<0.0001
51–90 min	262 (33%)	128 (38%)	134 (29%)	
90+ min	45 (6%)	27 (27%)	18 (4%)	
Duration^d^				
< 51 min	475 (60%)	172 (51%)	303 (66%)	<0.0001
51–90 min	262 (33%)	128 (38%)	134 (29%)	
90+ min	45 (6%)	27 (8%)	18 (4%)	
LOS After^e^				
≤ 7 days	654 (83%)	255 (76%)	399 (87%)	<0.0001
8–9 days	52 (7%)	29 (9%)	23 (5%)	
10+ days	86 (11%)	51 (15%)	35 (8%)	

^a^ *ASA* American Society of Anesthesiologists

^b^ *LOS Before* length of stay before the operation

^c^ *Total duration* total duration from admission to the operating room to end of surgery

^d^ *Duration* duration of the actual operation

^e^ *LOS After*, Length of stay after the operation

The time interval between hospital admission to surgery was significantly reduced in the post-OHSS patients compared to the pre-OHSS group: 42% vs. 33% of patients were operated within the first 24 h, and 38% vs. 20% within 24 to 48 h respectively, with a *p*-value of <0.0001. Patients in the post-OHSS period had a shorter operation time (both total and actual surgical duration with *p* values of < 0.0001) and a reduced post-operative length of stay compared to patients in the pre-OHSS group (Table 1).

**Fig. 1:**
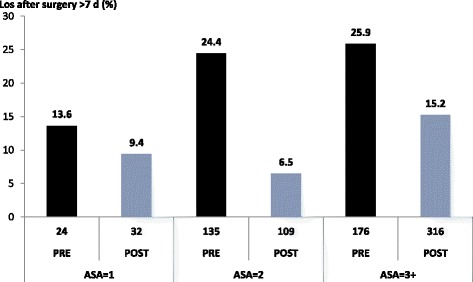
Multivariate analysis adjusting for ASA score comparing pre-OHSS and post-OHSS in length of stay (LOS) >7 days

Bivariate analysis for post-operative length of stay longer than 7 days showed that the variables that were associated with this length of stay after surgery were duration of the surgery itself and length of stay before the operation. Figure 1 illustrates the comparison between pre-OHSS and post-OHSS for post-operative hospitalization of more than 7 days, once ASA classification was adjusted.

**Fig. 2:**
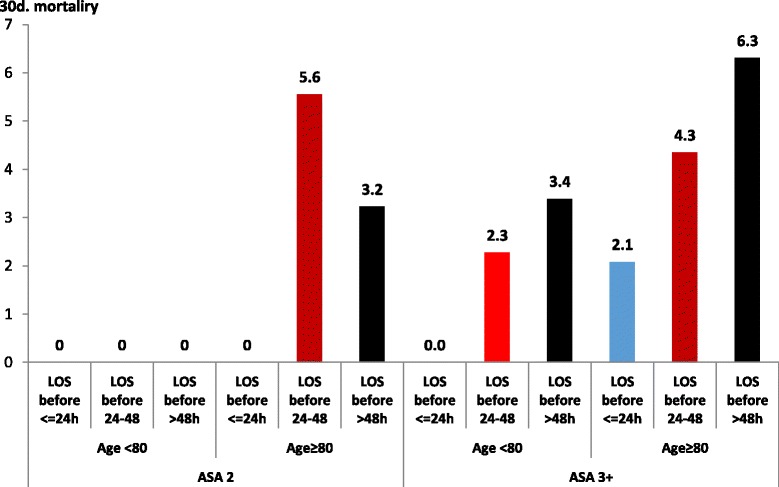
Multivariate analysis adjusting for ASA score comparing pre-OHSS and post-OHSS in 30 day mortality of patients

Bivariate analysis showed that the variables associated with post-operative mortality (within 30 days) were age, taking three medications or more, number of illnesses, ASA physical status classification of 3–4, length of stay before the operation and length of stay longer than 10 days after the operation. The ASA physical status classification takes into account the patient’s age. An ASA classification of 1 was found to be irrelevant for mortality, and therefore was not used in that multivariate analysis. Figure 2 illustrates the comparison between pre-OHSS and post-OHSS, once ASA and age was adjusted for in the multivariate analysis for mortality.

**Fig. 3:**
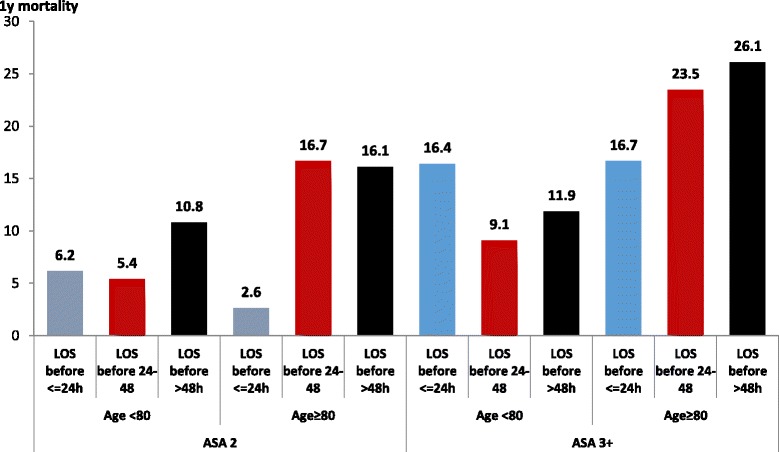
One year mortality of patients 80 years of age or more

**Fig. 4:**
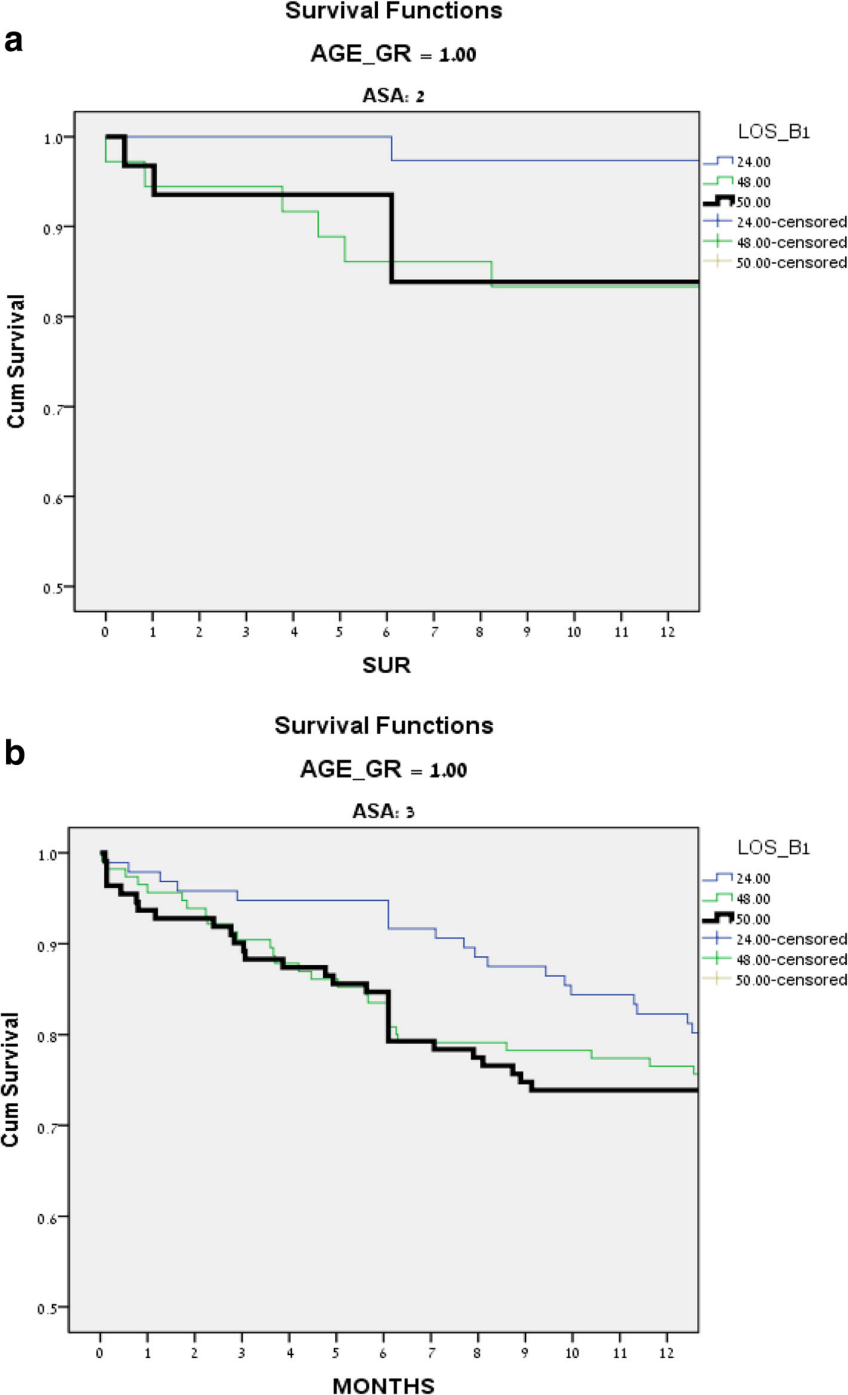
One year follow-up on post-operative survival (patients 80 year old or more). **a** ASA 2; **b** ASA 3

Older patients, 80 years or more, with higher ASA classifications were found to benefit the most from early operation and treatment. Figure 3 illustrates the 1 year mortality of 80-year old patients. The post-operative follow-up of 1 year survival of patients 80 years old or older is demonstrated in Figure 4. Patients 80 years or older, ASA 2, 3 or more, had significantly better 1 year survival if operated within 24 h. Patients in this age group had similar survival if operated after 24 to 48 h, or more.

The operation was delayed for more than 48 h in 100 patients in the pre-OHSS group, and in 48 patients in the post-OHSS group. Causes for the delay are summarized in Table 2. The groups are similar in terms of medical problems that caused the delay, and the main difference between them is the operating room availability.

**Table 2:** Data regarding the causes for delaying the operation for more than 48 h

Cause of the delay	Number of Patients	
	Pre-OHSS (*N* = 100)	Post-OHSS (*N* = 48)
Coagulation problem	12	15
Deterioration in cardiac status	8	8
Fever	2	3
Respiratory deterioration	2	1
Acute renal failure	1	1
New onset atrial fibrillation	0	2
Anemia	1	0
New onset neurologic symptoms	1	2
Operating room is not available	57	2
No explanation in the file	16	14

## Discussion

In this retrospective study we were able to show that, following implementation of the OHSS policy, the post-OHSS group had significantly reduced time to surgery in relation to the pre-OHSS group (42% vs. 33% in the first 24 h, and 38% vs. 20% within 24 to 48 h, *p* <0.0001). In addition, patients in the post-OHSS group were found to have significantly shorter length of stay post-operation (87% vs. 76% within the first 7 days, *p* < 0.0001). This supports the rationale for OHSS implementation, which states that by decreasing waiting time for an emergent hip fracture fixation we may reduce post-operative and total length of stay in the hospital and reduced mortality rate. Similar results were found by Peleg and colleagues, who analyzed data from several hospitals, and concluded that the OHSS reform was successful in decreasing the longer-term patient mortality after hip fracture [25]. Since the OHSS policy is still running in Israel public health system, and its execution is expensive, its validation is of consequence.

Another important finding in this study was that higher risk patients, with higher ASA physical status classification, were included in the post-OHSS group, comparing to the pre-OHSS patients (53% vs. 42% with ASA score 3, and 16% vs. 10% with ASA score 4, *p* < 0.0001). As previous studies have noted, higher ASA scores resulted in higher mortality and longer lengths of stay in the hospital post-operation [26–28]. Thus, we used a multivariate analysis to adjust for the ASA classification. After the adjustment, the post-OHSS group was found to have significantly decreased post-operative mortality.

With increasing ASA classification, the odds ratio increased as well: ASA physical status classification of 3 was associated with an odds ratio of 2.02, while ASA physical status classification of 4 was associated with an odds ratio of 3.23. This shows that patients with a higher ASA classification benefitted the most from the implementation of OHSS in terms of post-operative mortality. This revelation may have practical application, as the higher risk patients may benefit the most from a ‘fast-track’ course into surgery.

The inclusion of higher risk patients in the post-OHSS era may be open to discussion. This occurrence may be explained by the financial incentive both for the institution and the stuff, to operate the patient. This financial issue is a principal part of the OHSS policy. This may influence the judgment of the administrators and the caretakers into widening the boundaries and include more and more patients in the OHSS program. However, the global tendency is to operate older and older patient in worse health status, as a result of improved surgical and anesthetic capability and better-quality post-operative management and facilities [29, 30]. The downside of the OHSS policy, as with every financial-supported health care system, is the risk of abuse, meaning over-treatment. Patients who could benefit from conservative, non-surgical treatment, may be operated for the money. In addition, patients who require pre-operative preparation that takes more than 48 h, may not get it. The negative effect of the financial incentive on the medical management of patients is known [31, 32]. The question is whether the incentive should be paid for the treatment or for the outcome, for example: pay for complication-free post-operative period. How to move towards value-based purchasing is yet to be established.

Surgery was delayed due to medical causes in 27 patients and 32 patients in the pre- and post-OHSS period, respectively. Delaying surgery for more than 48 h due to administrative reasons occurred in 57 and 2 patients in the pre- and post-OHSS respectively. The reason for that delay was the unavailability of operating room and operating room personnel at the time needed. This demonstrates the valuable and effective consequence of OHSS policy on operating room management and thus on patients’ health and outcomes.

There are several limitations to this study, as it is a retrospective observational study. However, most of the studies published in this subject are retrospective [15, 17]. The pre-OHSS took place 2 years before the post-OHSS. Comparing the outcomes of procedures that were conducted in different years may be problematic, since medical and surgical care changes all the time; we hope for better, and improvement over time is to be expected. However, better OR management of personnel and surgical time may significantly affect post-operative outcomes. Another study weakness that originates from the fact that this is a retrospective study: there is a difference in group size: 335 patients in the pre-OHSS period and 457 in the post-OHSS period.

## Conclusion

In conclusion, in this study we showed that the implementation of the Out of Hours Surgery Service has led to a shorter period of waiting before surgery, decreased length of stay of patients post hip fracture surgery in the hospital, and decreased post-operative mortality. Adjusting for the ASA score of the patients allowed for this conclusion to be appreciated. Furthermore, the patients who benefitted the most from the OHSS policy were those with higher ASA classification, i.e. the older and sicker patients. More research is to be conducted to evaluate the disadvantages of financial-incentive effect on health care system and how to move towards value-based purchasing.

## Abbreviations

ASA: American society of anesthesiologists
CI: Confidence interval
LOS: Length of stay
OHSS: Out of hours surgery service
OR: Odds ratio
SD: Standard deviation
